# *Tribulus terrestris* L. Extract Protects against Lipopolysaccharide-Induced Inflammation in RAW 264.7 Macrophage and Zebrafish via Inhibition of Akt/MAPKs and NF-*κ*B/iNOS-NO Signaling Pathways

**DOI:** 10.1155/2021/6628561

**Published:** 2021-02-12

**Authors:** Wai-Rong Zhao, Wen-Ting Shi, Jing Zhang, Kai-Yu Zhang, Ye Qing, Jing-Yi Tang, Xin-Lin Chen, Zhong-Yan Zhou

**Affiliations:** ^1^Longhua Hospital, Shanghai University of Traditional Chinese Medicine, Shanghai, China; ^2^State Key Laboratory of Quality Research in Chinese Medicine and Institute of Chinese Medical Sciences, University of Macau, Macao, China

## Abstract

Inflammation response is a regulated cellular process and excessive inflammation has been recognized in numerous diseases, such as cardiovascular disease, neurodegenerative disease, inﬂammatory bowel disease, and cancer. *Tribulus terrestris* L. (TT), also known as Bai Jili in Chinese, has been applied in traditional Chinese medicine for thousands of years while its anti-inflammatory activity and underlying mechanism are not fully elucidated. Here, we hypothesize *Tribulus terrestris* L. extract (BJL) which presents anti-inflammatory effect, and the action mechanism was also investigated. We employed the transgenic zebrafish line Tg(MPO:GFP), which expresses green fluorescence protein (GFP) in neutrophils, and mice macrophage RAW 264.7 cells as the *in vivo* and *in vitro* model to evaluate the anti-inflammatory effect of BJL, respectively. The production of nitric oxide (NO) was measured by Griess reagent. The mRNA expression levels of inflammatory cytokines and inducible nitric oxide synthase (iNOS) were measured by real-time PCR, and the intracellular total or phosphorylated protein levels of NF-*κ*B, Akt, and MAPKs including MEK, ERK, p38, and JNK were detected by western blot. We found that BJL significantly inhibited fin transection or lipopolysaccharide- (LPS-) induced neutrophil migration and aggregation in zebrafish *in vivo*. In mice macrophage RAW 264.7 cells, BJL ameliorated LPS-triggered excessive release of NO and transcription of inflammatory cytokine genes including tumor necrosis factor-alpha (TNF-*α*), interleukin-6 (IL-6), and interleukin-1 beta (IL-1*β*). BJL also reduced the LPS-induced elevations of intracellular iNOS and nuclear factor kappa B (NF-*κ*B) which mediate the cellular NO and inflammatory cytokine productions, respectively. Moreover, LPS dramatically increased the phosphorylation of Akt and MAPKs including MEK, ERK, p38, and JNK in RAW 264.7 cells, while cotreatment BJL with LPS suppressed their phosphorylation. Taken together, our data suggested that BJL presented potent anti-inflammatory effect and the underlying mechanism was closely related to the inhibition of Akt/MAPKs and NF-*κ*B/iNOS-NO signaling pathways.

## 1. Background

Inflammatory response defenses the foreign attacks including tissue injury, pathogens, infections, and irritants and restores normal tissue function [1]. It plays a pivotal role in the physiological process of immunomodulation [2], and excessive inflammation response is also the main pathological feature in numerous chronic diseases, including cardiovascular disease, neurodegenerative disease, inﬂammatory bowel disease, rheumatoid arthritis, and cancer [3]. Many types of inflammatory cells, such as neutrophils, eosinophils, macrophages and mononuclear phagocytes, are involved in the inflammatory response [1]. The blood resident neutrophil is commonly the primary cell that migrates to the inflammatory position and initiates inflammatory action [4]. It is well-known that macrophages interact with neutrophils and clean the damage tissue and the other inflammatory cells by phagocytosis and consequently suppress the inflammation action [5, 6]. However, macrophages produce proinflammatory cytokines, such as TNF-*α*, IL-6, and IL-1*β*, which mediate by the transcript factor NF-*κ*B during the tissue repairing process [7, 8]. The production of NO is an important feature of inflammatory response mediated by macrophages, which causes cell oxidative damage and is dramatically regulated by iNOS [9]. The transcription of iNOS gene is regulated by various transcript factors including NF-*κ*B, activator protein-l (AP-l), interferon regulatory factor 1 (IRF1), and signal transducer and activator of transcription 1 (STAT1) [10]. Moreover, the activation of Akt/MAPKs signaling cascade was considered as one of the important physiological procedures on LPS-induced inflammation both in macrophage and zebrafish [11, 12]. Thus, pharmacological inhibition of Akt/MAPKs and NF-*κ*B/iNOS-NO signaling pathways might be effective for the suppression of tissue injury during inflammatory response.

*Tribulus terrestris* L. (TT, Zygophyllaceae family), which contains active components including alkaloids, steroidal saponins, ﬂavonoids, tannins, amino acids, quinines, and phenolic compounds, is a commonly used traditional Chinese herbal medicine, also known as Bai Jili in Chinese, for treating various diseases including hypertension, edema, eye problems, sexual dysfunction, and rheumatoid arthritis in clinics for thousands of years [13, 14]. The prosexual, cardiac-protective, muscle protective, neuroprotective, and osteoprotective effects of TT were most studied in the recent years [14–19], and the anti-inflammatory effect of TT might contribute to these broad range of biological effects [13]. In our previous studies, we have established multiple drug screening models using zebrafish, such as proangiogenesis [20, 21], antiangiogenesis [22], cerebral hemorrhage [23, 24], and neuroprotection [25, 26]. Zebrafish also has been supposed as one of the wildly used anti-inflammatory drug screening *in vivo* models, with several advantages including low cost, easy observation, and short test period [4, 27, 28]. In the present study, we evaluated the anti-inflammatory effect of BJL in zebrafish *in vivo* and mice macrophage RAW 264.7 cells *in vitro*, and the action mechanisms were also partially elucidated.

## 2. Materials and Methods

### 2.1. Ethic Statement

Zebrafish were kindly provided by Prof. Simon Lee from University of Macau and maintained by the Laboratory Animal Service Center, Longhua Hospital, Shanghai University of Traditional Chinese Medicine. All experiments were in accordance with the Longhua Hospital-Animal Experimentation Ethics Committee of Shanghai University of Traditional Chinese Medicine.

### 2.2. Chemicals

Lipopolysaccharide (LPS) was bought from Sigma Aldrich (St. Louis, USA). Nitric oxide (NO) detection kit was supplied by Beyotime Biotechnology (Shanghai, China). Primary and secondary antibodies were bought from Cell Signaling Technology (MA, USA). The whole plant of *Tribulus terrestris* L. (TT) was identified by the Pharmacy Department, Longhua Hospital. *Tribulus terrestris* L. extract (BJL) was prepared by Shanghai Institute of Materia Medica, Chinese Academy of Sciences (Shanghai, China). Briefly, TT was dried and smashed. 100 g TT powder was boiled within 1200 ml distilled water for 1 h and the supernatant was collected. The TT powder was continually boiled with 800 ml distilled water for 1 h and the supernatant was collected. These two supernatants were combined and stored at 5–10°C for 12 h. Then, the crude TT extract was filtered and absorbed by macroporous resin (1400, 2 : 1). Finally, the macroporous resin was washed by 60% ethanol, and the eluate was concentrated and sprayed to avoid dehydration. The yield of TT extract (BJL) was 4.92%. The chemical profile of BJL was analyzed by high performance liquid chromatography (HPLC) and is presented in supplementary materials (Figure S1). BJL was dissolved in dimethyl sulfoxide (DMSO) while other drugs were prepared in MiliQ water.

### 2.3. Zebrafish Maintenance and Morphological Observation

The transgenic zebrafish line Tg (MPO:GFP), which expresses green fluorescence protein (GFP) in neutrophils [29], was employed for anti-inflammatory activity evaluation. Zebrafish were maintained under a stander condition according to the zebrafish book (A guide for the laboratory use of zebrafish *Danio* (Brachydanio) *rerio*, by Monte Westerfield, Institute of Neuroscience, University of Oregon). Zebrafish embryos were generated by natural breeding and culture in embryo media at 28.5°C in an incubator. Three DPF (day post fertilization) zebrafish embryos were distributed in a 12-well plastic plate with 10 embryos in each group. On the fin transection model, zebrafish embryos were treated with various concentrations (3, 10, and 30 *µ*g/ml) of BJL for 2 h, and then zebrafish embryos were cut fins using a sharp needle. Then, zebrafish embryos were incubated with BJL for another 2 h, and the number of fluorescent cells migrated to wound was observed under a fluorescent stereoscopic microscope (Nikon SMZ18, Japan). On the LPS-injection model, zebrafish embryos were injected with LPS (0.3 *µ*g/ml), and then zebrafish embryos were treated with various concentrations (3, 10, and 30 *µ*g/ml) of BJL for 24 h. The fluorescence intensity of injection site in each embryo was calculated by Image J.

### 2.4. Cell Viability and Nitric Oxide (NO) Release Assay in RAW 264.7 Macrophage

The mice macrophage RAW 264.7 cell line was purchased from the American Type Culture Collection (ATCC) and cultured in Dulbecco's modified Eagle's medium (DMEM) supplemented with 10% FBS and 1% penicillin/streptomycin at 37°C in a humidified atmosphere of 5% CO_2_ in air. RAW 264.7 cells were seeded in a 96-well plate with a density of 2 × 10^4^ cells/well and further cultured for 24 h. RAW 264.7 cells were treated LPS (0.3 *µ*g/ml) with various concentrations (3, 10, and 30 *μ*g/ml) of BJL for 24 h. The cell viability was measured by MTT assay. The supernatant medium was used for NO release assay by total NO detection kit (Griess reagent, Beyotime Biotechnology). The results were presented as the percentage or folds of the control group.

### 2.5. Western Blot Analysis

RAW 264.7 cells were seeded in 60 mm × 15 mm Petri dish with a density of 5 × 10^5^ cells/dish and incubated for 24 h and then treated BJL (30 *μ*g/ml) with or without LPS (0.3 *μ*g/ml) for 24 h. After drug treatment, the RAW 264.7 cells were washed with ice cool PBS 3 times and lysed by RIPA buffer (Beyotime Biotechnology) on ice. The cell samples were collected and centrifuged (12000 g) for 15 min at 4°C. The supernatant of each sample was transferred to a new ice cool tube and its protein concentration was determined by BCA kit (Thermo Fisher). Each sample was denatured for 5 min at 95°C after adding the 5 × loading buffer (Beyotime Biotechnology). 30 *μ*g protein of each sample was applied for the protein separation by SDS-PAGE electrophoresis (Bio-Rad). Then, the separated protein was transferred to a PVDF membrane (0.22 *μ*m) by the semidry transfer system (Bio-Rad). The membrane was blocked by 5% skim milk in PBST for 2 h and then followed by primary antibodies including NF-*κ*B, Akt, Phospho-Akt, ERK1/2, Phospho-ERK1/2, p38, Phospho-p38, JNK, Phospho-JNK, and GAPDH (1 : 1000, Cell Signaling Technology) incubation over night with gentle shacking at 4°C. The membrane was washed with PBST 3 times and incubated with HRP-linked secondary antibody for 2 h at room temperature. Finally, the membrane was washed with PBST 3 times and imaged by the AM600 image system (GE Healthcare) after adding the ECL chemiluminescence substrate (Bio-Rad). The intensity of each band was calculated by Image J.

### 2.6. Real-Time PCR Analysis

RAW 264.7 cells were seeded in 60 mm Petri dish with a density of 5 × 10^5^ cells/well and incubated for 24 h at 37°C in a humidified atmosphere of 5% CO_2_ in air. RAW 264.7 cells were treated BJL (30 *μ*g/ml) with or without LPS (0.3 *μ*g/ml) for 24 h. The total RNA of each group was extracted by the TriPure Isolation Reagent (Roche, Manneheim, Germany) and their RNA concentrations were detected and calculated by absorbance at 260 nm using Microplate Reader (Tecan M200, NanoQuant). The first stranded cDNA was synthesized by cDNA synthesis kit (Roche, Manneheim, Germany). Finally, the mRNA expression level of interested gene was detected by real-time PCR with specific primers (Table 1) under the SYBR green-based real-time PCR system (Roche, LC96). The mRNA expression level of each gene was calculated by 2^−∆∆Ct^ relative quantification method with 3 independent replicates.

### 2.7. Data Analysis

Data were presented as the mean ± S.E.M. from at least three independent experiments. The student's paired *t*-test or analysis of variance (ANOVA) was used for statistical evaluation of difference between two groups. *p* < 0.05 indicated a significant difference.

## 3. Results

### 3.1. BJL Reduced LPS-Induced Excessive NO Release in RAW 264.7 Cells

**Figure 1:**
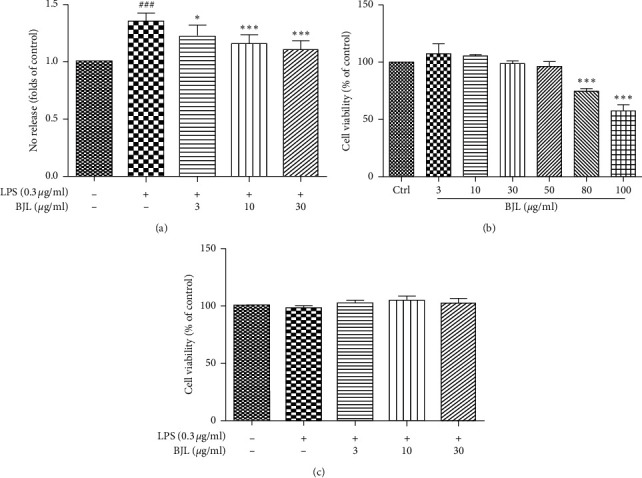
The cytotoxicity of BJL and BJL-decreased LPS-induced NO release in RAW 264.7 cells. (a) RAW 264.7 cells were treated LPS (0.3 *μ*g/ml) with or without various concentrations of BJL (3, 10, and 30 *μ*g/ml) for 24 h and then followed by NO release assay. (b) RAW 264.7 cells were treated with various concentrations of BJL (3, 10, 30, 50, 80, and 100 *μ*g/ml) for 24 h and the cell viability was measured by MTT assay. (c) RAW 264.7 cells were treated LPS (0.3 *μ*g/ml) with or without various concentrations of BJL (3, 10, and 30 *μ*g/ml) for 24 h and the cell viability was measured by MTT assay. Data were presented as the percentages of the control group. Results were means ± S.E.M. of more than 3 independent experiments. ^###^*p* < 0.001 versus the control group. ^*∗*^*p* < 0.05, ^*∗∗*^*p* < 0.01, and ^*∗∗∗*^*p* < 0.001 versus the LPS-treated group.

Nitric oxide (NO) production by macrophage is an essential process of inflammatory action. We found that LPS significantly promoted the NO production in mice macrophage RAW 264.7 cells, and BJL (3, 10, and 30 *μ*g/ml) concentration dependently inhibited the LPS-induced NO release (Figure 1(a)). As a result from the cell viability assay, BJL did not cause any cytotoxicity in Raw 264.7 cells at 50 *μ*g/ml (Figure 1(b)) and cotreatment BJL with LPS also did not cause any cytotoxicity at the indicated concentrations (Figure 1(c)). So, we could conclude that BJL reduced LPS-induced excessive NO release which was not associated with the cytotoxicity of BJL in RAW 264.7 cells.

### 3.2. BJL Ameliorated Fin Transection or LPS-Induced Migration and Aggregation of the Neutrophils in Zebrafish

**Figure 2:**
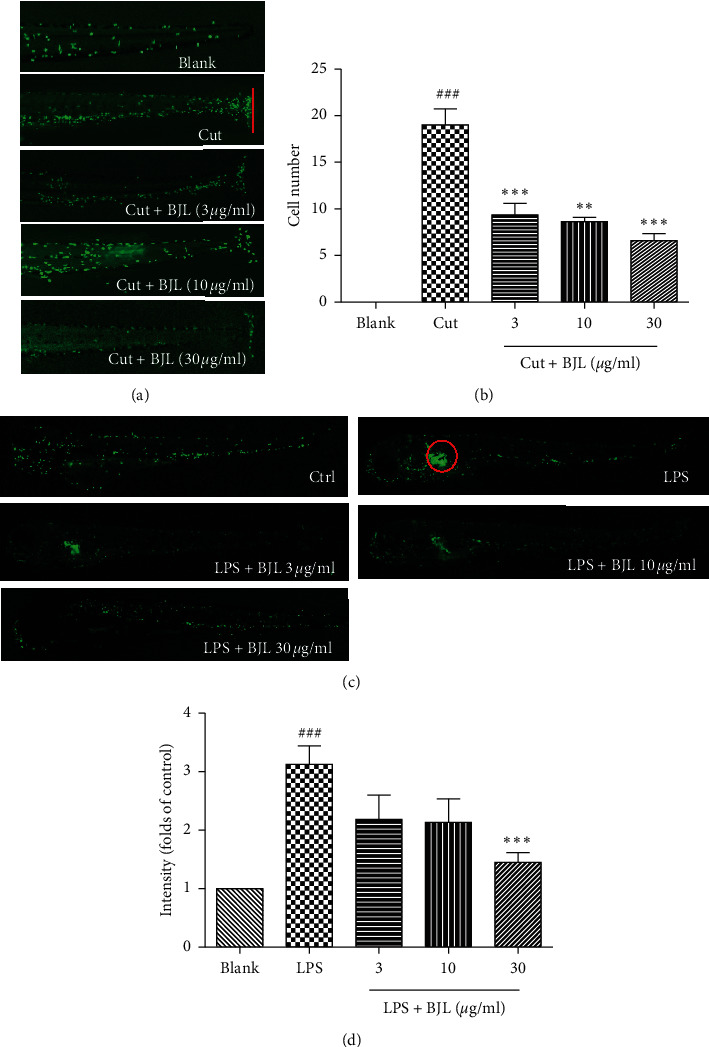
BJL ameliorated fin transection or LPS-induced migration and aggregation of the neutrophils in zebrafish. (a-b) The transgenic zebrafish line Tg(MPO:GFP) which expresses green fluorescence protein in neutrophils was employed in this experiment. Three DPF zebrafish embryos were treated with various concentrations of BJL (3, 10, and 30 *μ*g/ml) for 2 h and then zebrafish embryos were cut a part of the fin using a sharp needle and treated with BJL for another 2 h The photos were taken under a fluorescent stereoscopic microscope. The red line indicates the amputation site. The migrated cell numbers of neutrophils were calculated in each zebrafish embryo. (c-d) Three DPF zebrafish embryos were injected with LPS (0.3 *μ*g/ml) and then treated with various concentrations (3, 10, and 30 *μ*g/ml) of BJL for 24 h. The red circle indicates the injection site. The fluorescence intensity of the injection site was calculated by Image J data which were presented as the folds of the control group. Results were the means ± S.E.M. of more than 3 independent experiments. ^#^*p* < 0.05 and ^###^*p* < 0.001 versus the blank control group. ^*∗∗*^*p* < 0.01 and ^*∗∗∗*^*p* < 0.001 versus the LPS-treated group.

The migration and aggregation of neutrophils initiated the inflammatory action. In this study, we employed the transgenic zebrafish line Tg(MPO:GFP) which expressed green fluorescent protein in neutrophils to evaluate the anti-inflammatory effect of BJL. We found that fin transection significantly increased the cell number of the neutrophils which migrated and aggregated to the wound, and BJL (3, 10, and 30 *μ*g/ml) concentration dependently decreased these cell numbers in the amputation site (Figures 2(a) and 2(b)). Consistently, injection of LPS (0.3 *μ*g/ml) dramatically increased the fluorescent intensity on the injection site in zebrafish, which indicated cell number of the neutrophils increased on the injection site, and treatment BJL (3, 10, and 30 *μ*g/ml) with LPS (0.3 *μ*g/ml) reduced the fluorescent intensity (Figures 2(c) and 2(d)). These results suggested that BJL could inhibit the migration and aggregation of neutrophils during the inflammatory process.

### 3.3. BJL Partially Inhibited LPS-Induced Upregulation of NF-*κ*B, iNOS, and Inflammatory Cytokines including TNF-*α*, IL-6, and IL-*β* in RAW 264.7 Cells

**Figure 3:**
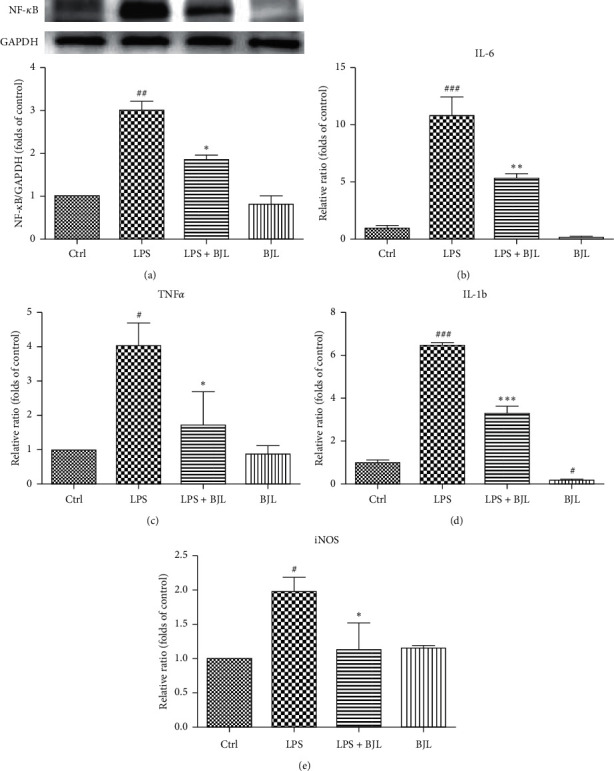
BJL partially inhibited LPS-induced upregulations of NF-*κ*B, iNOS, and proinflammatory cytokines in RAW 264.7 cells. RAW 264.7 cells were treated LPS (0.3 *μ*g/ml) with or without BJL (30 *μ*g/ml) for 24 h. (a) The intracellular protein level of NF-*κ*B was tested by western blotting using the specific primary antibody. (b–e) The mRNA expression levels of TNF-*α*, IL-6, IL-1*β,* and iNOS were detected by real-time PCR. Data were presented as the folds of the control group. Results were means ± S.E.M. of more than 3 independent experiments. ^#^*p* < 0.055, ^###^*p* < 0.01, and ^###^*p* < 0.001 versus the control group. ^*∗*^*p* < 0.05, ^*∗∗*^*p* < 0.01, and ^*∗∗∗*^*p* < 0.001 versus the LPS-treated group.

NF-*κ*B plays an important role in the inflammatory action which initiates the transcription of inflammatory cytokines such as TNF-*α*, IL-6, and IL-1*β*. We found that LPS significantly increased the intracellular amount of NF-*κ*B, and cotreatment BJL with LPS decreased the intracellular NF-*κ*B (Figure 3(a)). Considering the results from Figures 3(b)–3(d), we found that LPS upregulated mRNA expressions of TNF-*α*, IL-6, and IL-1*β* genes, and BJL significantly suppressed LPS-induced mRNA expressions of these proinflammatory cytokine genes. In addition, LPS also upregulated the mRNA expression level of iNOS which mainly mediated the secretion of NO induced by LPS in RAW 264.7 cells (Figure 3(e)). Thus, we could conclude that BJL significantly reduced the elevations of inflammatory cytokines stimulated by LPS, which might be related to the suppression of the intracellular protein level of NF-*κ*B.

### 3.4. BJL Suppressed LPS-Induced Phosphorylation of Akt, MEK, ERK, P38, and JNK in RAW 264.7 Cells

In order to explore the action mechanisms underlying the inhibition effect of BJL on LPS-triggered expression of inflammatory factors, we examined the effect of BJL on the phosphorylation of the key proteins in Akt/MAPK signaling pathways which regulated the proinflammatory response [12]. We found that LPS dramatically stimulated the phosphorylation of Akt, MEK, ERK, P38, and JNK, while cotreatment BJL with LPS significantly suppressed their phosphorylation (Figures 3(a)–3(e)). Thus, the action mechanisms of the anti-inflammatory effect of BJL might be associated with the inhibition of Akt/MAPK signaling cascades.

## 4. Discussion

Inflammatory response is one of the vital cellular physiological processes which maintain normal tissue functions, and the anti-inflammatory modulation by phytomedicine is beneficial for suppression of tissue damage during inflammatory response. In the present study, we evaluated the anti-inflammatory effect of *Tribulus terrestris* L. extract (BJL) in both transgenic zebrafish line Tg (MPO:GFP) *in vivo* and mice macrophage RAW 264.7 cells *in vitro*, and the action mechanisms underlying the anti-inflammatory activity of BJL were also studied.

Nitric oxide (NO) is an essential modulator during inflammatory response and presents cytotoxicity to pathogenic microbes while also damages host tissue [30]. NO usually plays a protective role in cardiovascular disease and vascular endothelial function [31, 32]. Promotion of the generation and bioavailability of NO were important mechanisms underlying the protective effect of traditional Chinese medicine in cardiovascular disease with vascular endothelial dysfunction, for example, hypertension, hyperglycemia, hyperlipidemia, and atherosclerosis [33–35], whereas excessive NO generation commonly plays a harmful role in the inflammatory response in macrophages [30, 36]. In the present study, we found that LPS promoted the overproduction of NO in mice macrophage RAW 264.7 cells (Figure 1(a)) which mimicked the harmful role of NO during inflammation process. Modulation of the production and bioavailability of NO is one of the potent strategies for treatment of diseases involved in inflammation [37, 38]. We found that the abnormal elevated NO level induced by LPS was significantly decreased in the BJL-treated group (Figure 1(a)). As we all know that the cell number and cell viability affect the NO production, LPS and BJL did not present any cytotoxicity in the indicated concentrations (Figures 1(b) and 1(c)). Thus, BJL significantly inhibited LPS-induced excessive NO production regardless of its cytotoxicity in RAW 264.7 cells.

Zebrafish has been considered as one of the emerged promising animal models for drug discovery. In our previous studies, we established zebrafish disease models including angiogenesis, neuroprotection, and cerebral hemorrhage and found several active compounds isolated from traditional Chinese medicine [20–26]. In the present study, we employed transgenic zebrafish line Tg(MPO:GFP) which expresses specific green fluorescence protein in neutrophils as the *in vivo* model to evaluate the anti-inflammatory effect of BJL. We found that fin transection significantly induced the migration and aggregation of neutrophils on amputation site while the BJL-treated group dramatically decreased the numbers of neutrophils in a concentration-dependent manner (Figures 2(a) and 2(b)). Consistently, zebrafish embryos were injected with LPS and the intensity of green fluorescence on the injection site was significantly elevated (Figures 2(c) and 2(d)). However, treatment BJL with LPS injection reduced the fluorescence intensity on the injection site (Figures 2(c) and 2(d)). These results revealed that both fin transection and LPS injection significantly induced the migration and aggression of neutrophils, and treatment BJL reduced inflammatory response.

The secretion of NO induced by LPS was mainly mediated by iNOS in macrophages [9], and iNOS gene expression is also regulated by NF-*κ*B, which is a vital nuclear transcript factor in inflammatory response and initiates the transcription of inflammatory factors including TNF-*α*, IL-6, and IL-1*β* [12]. We found that LPS stimulated the elevation of intracellular NF-*κ*B in RAW 264.7 cells, and treatment BJL suppressed the LPS-induced NF-*κ*B increase (Figure 3(a)). Moreover, the mRNA expression levels of inflammatory cytokine genes including TNF-*α*, IL-6, and IL-1*β* were dramatically elevated by LPS stimulation, while treatment BJL ameliorated LPS-induced gene expression of inflammatory cytokines in RAW 264.7 cells (Figures 3(b)–3(d)). In macrophage, the upregulation of iNOS is a key character of proinflammation [39]. We found that the mRNA expression level of iNOS was significantly increased in the LPS-treated group, while that was significantly decreased by BJL treatment (Figure 3(e)). Thus, we concluded that BJL presented anti-inflammatory effect in macrophage RAW 264.7 cells, which was associated with the downregulation of NF-*κ*B and inhibition of mRNA expression of inflammatory factors including iNOS, TNF-*α*, IL-6, and IL-1*β*.

**Figure 4:**
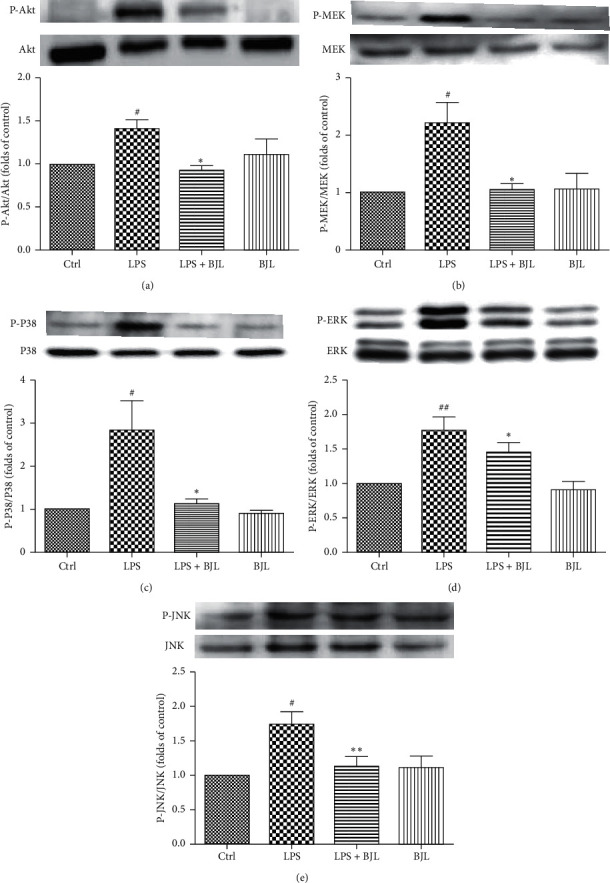
BJL suppressed LPS-stimulated activation of Akt/MAPK signaling cascades in RAW 264.7 cells. RAW 264.7 cells were treated LPS (0.3 *μ*g/ml) with or without BJL (30 *μ*g/ml) for 24 h and then followed by western blot analysis. Data were presented as the folds of the control group. Results were means ± S.E.M. of more than 3 experiments. ^#^*p* < 0.05 and ^##^*p* < 0.01 versus the control group. ^*∗*^*p* < 0.05 and ^*∗*^*p* < 0.01 versus the LPS-treated group.

LPS binds to the toll-like receptor (TLR) and stimulates the down-stream PI3K/Akt, MAPKs, and NF-*κ*B signaling cascades by the TIR domain-containing TLR signaling adapter BCAP (B-cell adapter for PI3K), which initiated inflammatory response [40, 41]. The inhibition of Akt/MAPKs is one of the essential mechanisms underlying the anti-inflammatory effect of natural products [42–44]. So, we proposed that the action mechanism underlying the anti-inflammatory effect of BJL was involved in Akt/MAPK signaling cascades. Considering the western blot results shown in Figure 4, LPS significantly increased the phosphorylation of Akt, MEK, ERK, P38, and JNK. Cotreatment BJL with LPS suppressed their phosphorylation in RAW 264.7 cells. Thus, the inhibition of Akt/MAPK signaling cascades was likely involved in the anti-inflammatory activity of BJL.

The anti-inflammation effect of *Tribulus terrestris* L. contributes to its effectiveness in multiple diseases while the anti-inflammatory component of *Tribulus terrestris* L. is not well-known. Lee et al. found that tribulusamide D, a compound isolated from *Tribulus terrestris* L., suppressed LPS-induced inflammatory response in RAW 264.7 cells via inhibition of P38 and NF-*κ*B signaling cascade [13]. N-trans-*ρ*-caffeoyl tyramine, which is also one of the components of *Tribulus terrestris* L., presented anti-inflammatory activity in LPS-stimulated RAW 264.7 cells by suppression of the protein levels of COX-2 and p-JNK [45]. So, these two compounds might be the active constituents of BJL in the present study.

## 5. Conclusion

**Figure 5:**
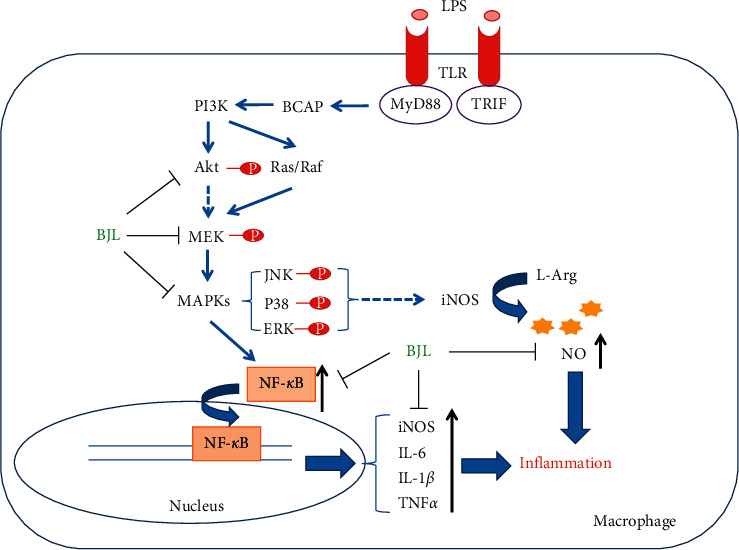
Schematic overview of the mechanism underlying the anti-inflammatory effect of BJL.

BJL presented anti-inflammatory effect both in zebrafish *in vivo* and macrophage *in vitro*, and the underlying mechanisms were associated with the inhibition of Akt/MAPKs and NF-*κ*B/iNOS-NO signaling cascades and the reduction of inflammatory factors including TNF-*α*, IL-6, and IL-1*β* (Figure 5). Thus, we could conclude that BJL was potential for developing as treatment agent for the treatment of diseases involved in inflammation, such as atherosclerosis, inﬂammatory bowel disease, and rheumatoid arthritis. In addition, the active component of BJL and the related animal study should be done in the future study.

## Figures and Tables

**Table 1:** Specific gene primers used in real-time PCR.

Accession number	Gene	Forward primer	Reverse primer
NM_013693.3	TNF-*α*	5′-TTC TCA TTC CTG CTT GTG G-3′	5′-ACT TGG TGG TTT GCT ACG-3′
NM_031168.2 NM_001314054.1	IL-6	5′-GAG GAT ACC ACT CCC AAC AGA CC -3′	5′-AAG TGC ATC ATC GTT GTT CAT ACA-3′
NM_008361.4	IL-1*β*	5′-AGA GCA TCC AGC TTC AAA T-3′	5′-CAT CTC GGA GCC TGT AGT G-3′
NM_001313922.1 NM_001313921.1 NM_010927.4	iNOS	5′-CATTGATCTCCGTGACAGCC-3′	5′-CATGCTACTGGAGGTGGGTG-3′
NM_008084.3 NM_001289726.1	GAPDH	5′-CCT TCC GTG TTC CTA CCC-3′	5′-CAA CCT GGT CCT CAG TGT AG-3′

## Data Availability

All the data used to support the findings of this study are available from the corresponding author upon reasonable request.

## Abbreviations

AP1: Activator protein-l
BCAP: B-cell adapter for PI3K
BJL: *Tribulus terrestris* L. extract
DPF: Day post fertilization
GFP: Green fluorescent protein
HPLC: High performance liquid chromatography
iNOS: Inducible nitric oxide synthase
IL-1*β*: Interleukin-1 beta
IL-6: Interleukin-6
IRF1: Interferon regulatory factor 1
LPS: Lipopolysaccharides
MAPKs: Mitogen-activated protein kinases
NF-*κ*B: Nuclear factor kappa B
NO: Nitric oxide
STAT1: Signal transducer and activator of transcription 1
TNF-*α*: Tumor necrosis factor-alpha
TLR: Toll-like receptor
TT: *Tribulus terrestris* L.

## Supplementary Materials

Supplementary Materials Figure S1: chemical profile analysis of BJL by high performance liquid chromatography (HPLC). .
