# Supplementary material for: Tribulus terrestris L. Extract Protects against Lipopolysaccharide-Induced Inflammation in RAW 264.7 Macrophage and Zebrafish via Inhibition of Akt/MAPKs and NF-κB/iNOS-NO Signaling Pathways
Source: Evid Based Complement Alternat Med. 2021 Feb 12;2021:6628561. doi: 10.1155/2021/6628561 (PMC7895590; doi:10.1155/2021/6628561)
Supplement: Supplementary Materials — Figure S1: chemical profile analysis of BJL by high performance liquid chromatography (HPLC). . [file 6628561.f1.docx]

**Supplemental data**


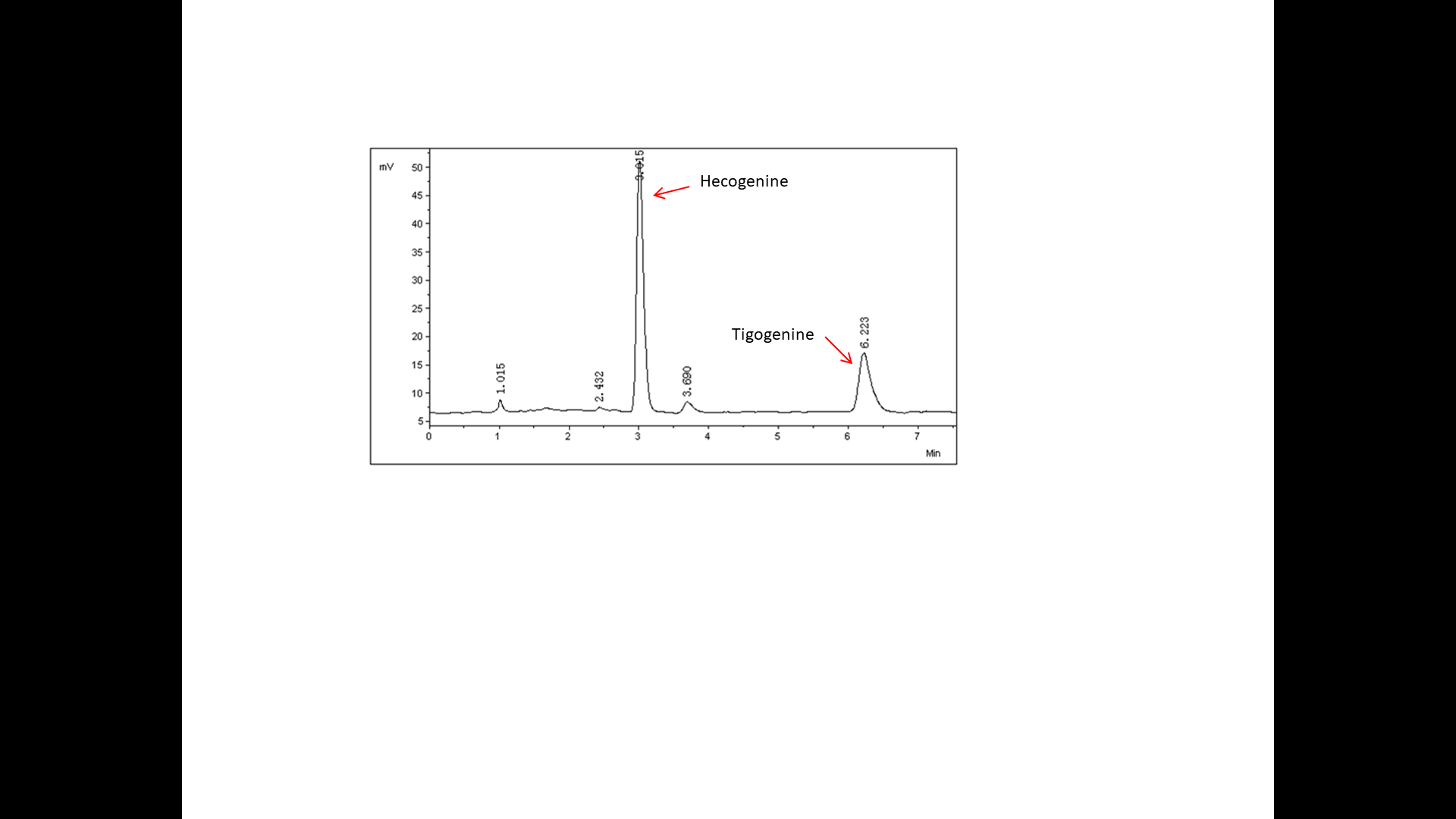


**Fig. S1 Chemical profile analysis of BJL by High Performance Liquid Chromatography (HPLC).** The BJL was hydrolysis by HCl in boiled water. Then the sample was cool down and filtered. The precipitate was washed with distill water until neutral. Then the chemical analysis of BJL was performed according to the stander method described in Chinese Pharmacopoeia (v2005, VI D).
